# Nanoanalytics for materials science

**DOI:** 10.3762/bjnano.7.159

**Published:** 2016-11-10

**Authors:** Thilo Glatzel, Tom Wirtz

**Affiliations:** 1 Department of Physics, University of Basel, Klingelbergstrasse 82, 4056 Basel, Switzerland; 2 Advanced Instrumentation for Ion Nano-Analytics (AINA), MRT Department, Luxembourg Institute of Science and Technology (LIST), 41 rue du Brill, L-4422 Belvaux, Luxembourg

With the progress in fabricating more energy efficient and sustainable devices, an increased need for advanced materials and processing techniques arises that becomes increasingly challenging and demands for new analysis techniques. In particular excellent spatial resolution together with high-sensitivity chemical information at the nanoscale are of utmost importance for future developments [1]. One of the possible approaches is based on bimodal or even multimodal nanoanalysis, which was increasingly developed during the last decade. Other important aspects that have to be considered are the impact of the applied measurement technique on the results, the time needed to get representative and repeatable results, and the possibility to apply the technique under relevant environmental conditions.

These nanoanalytical techniques need detailed understanding of the physical processes included in both the device structures and detection techniques. A typical setup includes a probe (such as tip, ion beam or electron beam), the condition of the sample and the interaction between them, which all need to be extensively investigated by simulations and modeling in order to obtain an in-depth and reliable understanding and accurate physical models. Furthermore, a reliable and easy way to extract a maximum of information out of the multimodal datasets, efficient data visualization strategies, and methods for analysis, mining and modeling are of utmost importance.

This Thematic Series on “Nanoanalytics for materials science” groups six exciting articles around the aforementioned aspects of nanoanalytics, describing the development of both new instrumentation as well as new methodologies.

On the side of instrumental development, a ultra-high resolution multi-probe device based on tuning fork sensors for both nanoindentation and depth sensing is presented by Cinar and co-workers [2]. Furthermore, Fleming et al. present an in situ combination of atomic force microscopy (AFM) and secondary ion mass spectrometry (SIMS) [3]. By doing so, they obtain high-resolution 3D elemental/chemical maps. This approach delivers not only complementary information but furthermore allows for determining and minimizing artefacts occurring in standard SIMS 3D analysis, as for example disturbed height information induced by inhomogeneities of the sputter rates (caused by samples containing various materials, different phases or having a non-flat surface) and varying secondary ion extraction efficiencies due to local field distortions (caused by topography with high aspect ratios).

An ex situ multi-modal approach combining transmission electron microscopy (TEM) as well as X-ray and IR spectroscopies was successfully applied to investigate magnetite nanoparticles by Kalska-Szostka et al. [4]. TEM was also used in the work of Gutsch et al. who developed a novel energy-filtered transmission electron microscopy (EFTEM) approach using ultrathin TEM membranes [5]. With this method, they were able to accurately study the morphology of Si nanocrystal ensembles without the limitations imposed by standard cross sectional TEM investigations.

Advances in preparing high-performance probes for magnetic force microscopy (MFM), with a particular focus on controlling and tuning the tip stray field, are presented by Iglesias-Freire and co-workers [6]. Finally, Mirzaei and Kiani described the vibration behavior of carbon-nanotube-reinforced composite structures highlighting the importance but also the feasibility of theoretical approaches in complex nanoanalytical studies [7].

Finally, we want to express our thanks to all authors for contributing their excellent work to this Thematic Series. We are also very grateful to the reviewers for their support by examining the papers and providing helpful reports, and the efficient and professional support by the editorial and production team of the *Beilstein Journal of Nanotechnology*.

Thilo Glatzel and Tom Wirtz

Basel and Belvaux, September 2016
